# ALDH2 Activity Reduces Mitochondrial Oxygen Reserve Capacity in Endothelial Cells and Induces Senescence Properties

**DOI:** 10.1155/2018/9765027

**Published:** 2018-11-14

**Authors:** G. Nannelli, E. Terzuoli, V. Giorgio, S. Donnini, P. Lupetti, A. Giachetti, P. Bernardi, M. Ziche

**Affiliations:** ^1^Department of Life Sciences, University of Siena, Siena, Italy; ^2^Department of Biomedical Sciences, University of Padua, Padua, Italy; ^3^Consiglio Nazionale delle Ricerche, Neuroscience Institute, Padua, Italy

## Abstract

Endothelial cells (ECs) are dynamic cells that turn from growth into senescence, the latter being associated with cellular dysfunction, altered metabolism, and age-related cardiovascular diseases. Aldehyde dehydrogenase 2 (ALDH2) is a mitochondrial enzyme metabolizing acetaldehyde and other toxic aldehydes, such as 4-hydroxynonenal (4-HNE). In conditions in which lipid peroxidation products and reactive oxygen species (ROS) are accumulated, ECs become dysfunctional and significantly contribute to the progression of vascular-dependent diseases. The aim of the present study has been to investigate whether inhibition of ALDH2 alters endothelial functions together with the impairment of bioenergetic functions, accelerating the acquisition of a senescent phenotype. HUVECs transfected with siRNA targeting ALDH2 or treated with daidzin, an ALDH2 inhibitor, were used in this study. We observed an alteration in cell morphology associated with endothelial dysfunctions. Loss of ALDH2 reduced cell proliferation and migration and increased paracellular permeability. To assess bioenergetic function in intact ECs, extracellular flux analysis was carried out to establish oxygen consumption rates (OCR). We observed a decrease in mitochondrial respiration and reserve capacity that coincided with SA-*β*-Gal accumulation and an increase in p21 and p53 expression in siALDH2 or daidzin-treated HUVECs. Treatment with N-acetyl-L-cysteine (NAC) reduced endothelial dysfunctions mediated by siALDH2, indicating that oxidative stress downstream to siALDH2 plays an instrumental role. Our results highlight that ALDH2 impairment accelerates the acquisition of a premature senescent phenotype, a change likely to be associated with the observed reduction of mitochondrial respiration and reserve capacity.

## 1. Introduction

The aging process reflects the age-dependent functional decline of body tissues and organs [1]. The progressive decline also affects the vascular endothelium, resulting in an impairment of its important functions such as the capacity to supply nutrients and growth factors to organs, the barrier function, and the capacity to form new vessels or angiogenesis [2, 3]. Vascular cell senescence is widely considered one of the causative factors of peripheral and central nervous system pathologies [4], as it promotes reactive oxygen species (ROS) production and the ensuing vascular inflammatory responses [5]. For instance, frequent stroke episodes in patients affected by cerebral amyloid angiopathy (CAA) are related to the perivascular deposition of amyloid beta peptides (A*β*), which promote ROS production and, in turn, reduce endothelial cell (EC) responsiveness. Similarly, in MELAS, a disease characterized by encephalomyopathy, high ROS levels, derived from dysfunctional mitochondria, compromise EC-mediated vasodilatation, which explains the susceptibility of these patients to stroke-like episodes [6–8]. Besides increased ROS production, nine hallmarks have been proposed to contribute to the aging phenotype, including the phenomenon of cellular senescence as a stable arrest of cell cycle coupled with phenotypic changes, mitochondrial dysfunction, and activation of the p53 pathway [1].

Even though ECs appear to meet most of their energy needs anaerobically, they have an extensive mitochondrial network and consume oxygen. Their mitochondria are essential to endothelial functions [9–11]. It is broadly accepted that, as cells are exposed to stressors, mitochondria are able to rely on a “reserve capacity,” referred to as the difference between the maximum respiratory capacity and the basal respiratory capacity. This reserve capacity is suitable to provide the increased energy demands to preserve cellular functions and repair or detoxification of reactive species [11, 12].

In this study, we sought to gain insights into the role of endothelial mitochondria in the senescence process, taking advantage of the recent progress gathered on the role of aldehyde dehydrogenases (ALDHs), particularly ALDH2 isozyme (one of 19 enzymes belonging to the same superfamily). The ALDH2 isozyme is located in the mitochondrial matrix and is predominantly responsible for the acetaldehyde detoxification in alcohol metabolism. It is also a key to detoxification of endogenous aldehydes, such as 4-hydroxy-2-nonenal (4-HNE), which arise from lipid peroxidation under oxidative stress, and of exogenous aldehyde products, such as acrolein from tobacco smoke and car exhausts [13, 14]. Increasing evidence has revealed a cardioprotective and neuroprotective role of ALDH2 in myocardial ischemia-reperfusion injury and A*β*-induced damage, respectively. Studies from our laboratory have provided evidence on the contribution of ALDH2 to A*β*-induced endothelial dysfunction and on the possibility of preserving the endothelial function in some pathological conditions by activating the ALDH2 enzyme [15]. In this study, we focused on ALDH2 and its role as a critical metabolic checkpoint for endothelial function. The aim of the study was to move beyond correlative analyses between ALDH2 and hallmarks of aging, providing causal evidence of the implication of ALDH2 in endothelial senescence.

## 2. Materials and Methods

### 2.1. Cell Cultures

Human umbilical vein endothelial cells (HUVECs) (Lonza, Basel, Switzerland) were used. Cells were cultured on gelatin-coated dishes with endothelial growth medium (EGM-2) (Lonza) supplemented with antibiotics (100 U/ml penicillin and 100 *μ*g/ml streptomycin, Euroclone, Milan, Italy), glutamine (2 mM, Euroclone), and 10% fetal bovine serum (FBS, Hyclone, GE Healthcare, Little Chalfont, UK). Progressively passaged HUVECs were utilized up to senescence as already described [16]. The formula PD = (ln *n*_ch_ − ln *n*_cs_)/ln 2 was used to calculate the number of cumulative population doublings (PD), where *n*_ch_ is the number of cells harvested and *n*_cs_ is the number of cells seeded.

### 2.2. Small Interfering RNA Transfection

Transient knockdown experiments were performed using control and specific siRNAs (OriGene, Rockville, MD, USA). Subconfluent cells were seeded in 60 mm or 100 mm or 6-well plate dishes in EGM-2 plus 10% FBS. After 24 h, cells were transfected in endothelial basal medium (EBM-2) without serum and antibiotics using Lipofectamine® 3000 (Invitrogen, Carlsbad, CA, USA) and 20 nM targeting siRNA (siALDH2) or scrambled control siRNA (siCTR) diluted in Opti-MEM (Gibco, Thermo Fisher Scientific, Waltham, MA, USA). Serum was added 6–8 h posttransfection. Where indicated, cells were harvested 24 h posttransfection. Cells were assayed 2–6 days posttransfection. The transfection was repeated every 72 h. We evaluated the knockdown efficiency using immunoblotting or quantitative RT-PCR (qRT-PCR) analysis at the indicated time.

### 2.3. Immunoblot Analysis

Subconfluent cells were plated in 60 mm or 100 mm or 6-well plate gelatin-coated dishes and transfected as above or treated with 10 *μ*M daidzin, for 48 h. Daidzin was dissolved in DMSO (Sigma-Aldrich). The treatment with daidzin was repeated every day. Where indicated, siCTR and siALDH2 HUVECs have been pretreated with 5 mM N-acetyl-L-cysteine (NAC), as reported [17]. Next, cells were washed and lysed, and an equal amount of proteins was used for immunoblot analysis, as described [15]. The blotted membranes were incubated with anti-ALDH2 (OriGene), anti-p21, anti-Egr-1, anti-c-Myc (Cell Signaling Technology, Danvers, MA, USA), anti-tubulin, anti-p53 (Santa Cruz, Heidelberg, Germany), anti-*β*-actin (Sigma-Aldrich), anti-4-HNE (Abcam, Cambridge, United Kingdom), and OXPHOS antibody cocktail kit (Abcam) antibodies. SuperSignal West Pico Chemiluminescent Substrate (Thermo Fisher Scientific) was used to develop signals, which were detected and quantified by the ChemiDoc system and Quantity One software (Bio-Rad, Hercules, CA, USA). For all experiments using whole-cell lysate, *β*-actin or *β*-tubulin was used as loading controls.

### 2.4. Real-Time PCR

The RNeasy Plus Kit (Qiagen, Hilden, Germany) was used according to the manufacturer's instructions to extract and prepare total RNA. A total amount of 1 *μ*g RNA was transcribed, and quantitative RT-PCR was performed as reported [18]. The fold change expression was determined using the comparative Ct method (ΔΔCt) normalized to 60S ribosomal protein L19 expression. Data are reported as fold change relative to siCTR (control), which was set to 1.

### 2.5. ALDH Enzymatic Activity

The conversion of acetaldehyde to acetic acid was determined in order to evaluate ALDH enzyme activity, as reported [15]. Briefly, 1 × 10^6^ of seeded cells were transfected as described above, and after 48 h, cells were scraped into 100 *μ*l of CelLytic™ MT Cell Lysis (Sigma-Aldrich) supplied with protease inhibitors (Sigma-Aldrich) and sodium orthovanadate (Sigma-Aldrich). Then, lysates were centrifuged for 20 min at 4°C at 14000 × *g*. The protein content in the supernatant was quantified in a Bradford assay. ALDH2 activity was measured in an assay mix (0.8 ml) containing 100 mM sodium pyrophosphate (Sigma-Aldrich) at pH 9 and 10 mM NAD+ (Sigma-Aldrich) and 300 *μ*g of sample protein. Then, acetaldehyde (10 mM, Sigma-Aldrich) was added to the cuvette to start the reaction. NADH formation from NAD+ was monitored at 25°C in a spectrophotometer Infinite F200 Pro at 340 nm (Tecan Life Sciences, Switzerland). Where indicated, supernatants of HUVECs were challenged with daidzin for 10 min before the acetaldehyde was added; the compound was tested at 1 and 10 *μ*M to monitor the extent of ALDH inhibition in these cell lysates. Enzyme-specific activity was expressed as % nmol NADH/minute/mg protein.

### 2.6. Cell Survival and Area

Cells were transfected with siRNA for ALDH2 as described above. Then, cells were harvested and seeded (1.5 × 10^3^/well) in triplicate in 96-multiwell plates. Adherent cells were exposed to EBM-2 with 2% FBS for 2 and 5 days. Where indicated, HUVECs were treated with 5 mM NAC (Sigma-Aldrich) before the administration of 2% FBS. This treatment was repeated every three days. Further, HUVECs were also pretreated for 30 min with 10 *μ*M daidzin in EBM-2 before the administration of 2% FBS for 2 or 5 days. Then, cells were fixed and stained with the PanReac kit (Darmstadt, Germany), and five fields per well were counted. Data are analyzed in triplicate, and results are expressed as the cell number counted/well. To calculate the area of cells, three fields in which cells were at 60% confluence for each condition were measured using ImageJ software and results are expressed as square pixel.

### 2.7. Senescence-Associated *β*-Galactosidase (SA-*β*-Gal) Activity Assay

Cells were seeded in 6-well multiplates (8 × 10^4^ or 1.5 × 10^5^ cells/well). Adherent cells were treated with 10 *μ*M daidzin for 2 days or transfected with siRNA as described above. Where indicated, 24 h posttransfection, a preincubation of 30 min with 5 mM NAC was carried out before the administration of 2% FBS. This treatment was repeated every three days. At 2 and 6 days, SA-*β*-Gal activity was assessed by using the Senescence *β*-Galactosidase Staining Kit (Cell Signaling) following the manufacturer's manual. Data are reported as a fold increase vs. siCTR of positive cells for SA-*β*-Gal activity.

### 2.8. BrdU Incorporation Assay

Cell proliferative capacity was evaluated using a chemiluminescence ELISA (Roche Diagnostic S.p.A, Monza, Italy) which assesses the 5-bromo-2′-deoxy-uridine (BrdU) incorporation. 3 × 10^3^ cells were seeded in a 96-well plate in triplicate 24 h posttransfection. Adherent cells were exposed to EBM-2 in the presence or absence of serum (2% and 0.1% FBS, respectively) for 48 h. 8 hours before the end of incubation, BrdU was added in each well. Then, the assay was carried out as reported [18].

### 2.9. Wound Healing Assay

siRNA transfection of adherent cells was conducted as described above, and after 24 h, cells were harvested. siCTR, siALDH2, and wild-type HUVECs were seeded (1 × 10^5^ cells/well) into 24-well plates and incubated until they were grown into a confluent monolayer (24–30 h). Then, a sterile 100–1000 *μ*l micropipette tip was used to scrape the confluent monolayer and create a wound ± 500 *μ*m. The cells were washed twice with PBS and exposed to fresh EBM-2 medium supplemented with 0.1 or 2% FBS and ARA-C (2.5 mg/ml, Sigma-Aldrich) to suppress cell proliferation. Where indicated, confluent HUVECs were pretreated for 30 min with daidzin and then treated with 2% FBS. Images of the wound in each well were acquired from 0 to 18 h under a phase contrast microscope at 10x magnification. Then, cells were fixed and stained with the PanReac kit. Results were quantified using ImageJ software, and data are reported as % of scratch closure.

### 2.10. Immunofluorescence Microscopy Analysis

Cells were transfected with siRNA as described above, and 24 h posttransfection, ECs were harvested and seeded (8 × 10^4^ cells) on 8 mm ø glass coverslips in triplicate. After 24 h, cells were exposed to EBM-2 with 0.1% FBS for 8 h. 4% paraformaldehyde/PBS with Ca^2+^ and Mg^2+^and 3% bovine serum albumin (BSA) were used to fix cells and block unspecific binding sites, respectively. Then, cells were incubated with a monoclonal rabbit anti-VE-cadherin diluted to 1 : 400 (Cell Signaling Technology) and a polyclonal rabbit anti-ZO-1 (Life Technologies, Carlsbad, CA, USA) diluted to 1 : 50 in 0.5% BSA in PBS for 18 h at 4°C. After incubation with the secondary antibody, Alexa Fluor© 488-labeled anti-rabbit (1 : 200, 1 h, Invitrogen) and Alexa Fluor© 555-labeled anti-rabbit (1 : 200, 1 h) were used for 1 h at room temperature, and then the protocol was completed according to Terzuoli et al. [19]. Leica SP5 confocal microscopy (63x objective) was used to capture images of stained cells.

### 2.11. Paracellular Permeability Assay

Cells were seeded (8 × 10^4^ cells/well) 24 h post-ALDH2 silencing on gelatin-coated insert membranes (0.4 *μ*m diameter pores, Corning, New York, USA), and the inserts were placed in 24-multiwell plates and incubated for 24 h. Next, cells were exposed to EBM-2 with 0.1% FBS for 8 h. The assay was carried out as described [20]. Briefly, the fluorescent permeability tracer (3 kDa FITC-Dextran, 10 *μ*M) was added, and the fluorescence was measured after 7 min in the medium present in the bottom of the well, in a multiplate reader (Infinite 200 Pro, SpectraFluor, Tecan), at 485/535 nm (excitation/emission). Results are reported as relative fluorescence units (RFU) [20].

### 2.12. ROS Measurement

siCTR and siALDH2 cells (24 h posttransfection) were seeded (1.5 × 10^3^ cells/well) in triplicate in a 96-well plate, and after adherence (6–8 h), the medium was replaced with EBM-2 with 0.1% FBS in the presence or absence of 5 mM NAC for 24 h. DCFH2-DA (2,-7-dichlorodihydrofluorescein diacetate, Invitrogen) was used, and intracellular ROS were measured as previously described [20]. The results are reported as fold change vs. siCTR of relative fluorescence units (RFU) corrected for the cell number counted [20].

### 2.13. Measurement of the Oxygen Consumption Rate

The measurement of oxygen consumption rates (OCR) was carried out using the Seahorse XF24 extracellular flux analyzer as described previously [21, 22]. siCTR and siALDH2 ECs were harvested and seeded 24 h posttransfection in XF24 cell culture plates at 3 × 10^4^ cells/well density in 200 *μ*l of EGM-2 supplemented with 10% FBS and incubated at 37°C in 5% CO_2_ for 6–8 h. Then, adherent cells were exposed to EBM-2 supplemented with 2% FBS for 24 h. Assays were performed as reported [22]. Independent titration was routinely carried out to determine the optimal concentration of FCCP (carbonylcyanide-p-trifluoromethoxyphenyl hydrazone), which was ranged between 0.2 and 0.4 *μ*M. Initially, a stable OCR baseline was determined, and then, oligomycin, FCCP, rotenone, and antimycin A were supplied as reported in the figure legend.

### 2.14. Transmission Electron Microscopy

1 × 10^4^ cells transfected with siRNA as above were harvested and seeded 24 h posttransfection in a small chamber prepared with cylinder part of BEEM® (Ted Pella Inc., Redding, CA, USA) capsule glued to the coverslip as previously described [23]. After 6–8 h of incubation needed for adhesion, cells were exposed to EBM-2 with 2% FBS for 24 h. The medium was replaced with 2.5% glutaraldehyde (Ted Pella, Redding, CA, USA) in 0.1 M phosphate buffer (pH 7.2) for 2 h at RT and postfixed with buffered 1% OsO_4_ (EMS, Hatfield, PA, USA) for 1 h and processed by standard dehydration through a graded series of ethanol (50°–100°). Specimens were then embedded in pure Epon (EMS, Hatfield, PA, USA) resin. Polymerization was done in an oven at 60°C for 48 h. Then, slides were dropped into liquid nitrogen to detach the resin from coverslips. 60–70 nm thick sections were cut with an Ultracut E (Reichert-Jung, Wien, AT) ultramicrotome, stained with uranyl acetate and lead citrate, and examined in a TEM Jeol 1010 (Peabody, MA, USA) transmission electron microscope.

### 2.15. Statistical Analysis

Results are expressed as means ± SD or SEM. Statistical analysis was generated by GraphPad software (San Diego, CA, USA). Statistical analysis was performed by Student's *t*-test and two-way ANOVA. *p* < 0.05 was considered statistically significant.

## 3. Results

### 3.1. ALDH2 Silencing or Inhibition Impairs Endothelial Cell Functions

To examine the role of ALDH2 in endothelial function, HUVECs were treated with 1 and 10 *μ*M daidzin, an inhibitor of ALDH2 activity [14], or transiently knocked down for ALDH2 (siALDH2) and compared with wild-type cells transfected with an empty vector (siCTR), throughout this work. 10 *μ*M daidzin was the higher concentration without a significant effect of its solvent on HUVEC survival.

**Figure 1:**
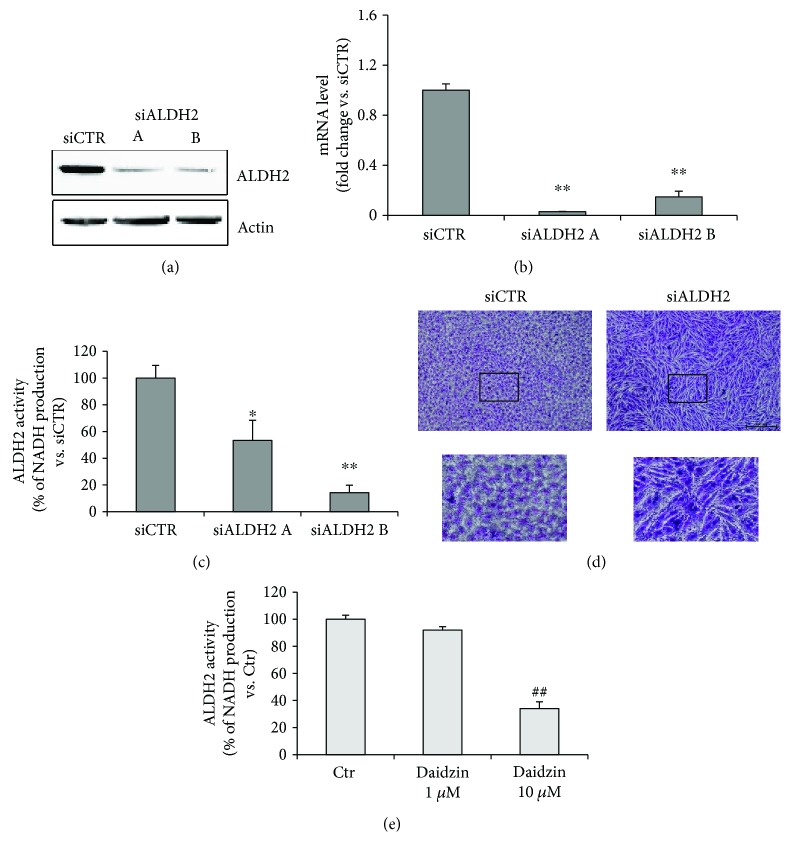
Characterization of siALDH2 and daidzin-treated HUVECs. (a) Immunoblotting and (b) qPCR analysis of ALDH2 silencing (siALDH2) in ECs, 48 h posttransfection. Representative blots of at least 3 with similar results are shown. qPCR data are expressed as the mean of fold change ± SD vs. siCTR cells, which were assigned to 1. ^∗∗^*p* < 0.01 vs. siCTR. (c) ALDH2 activity measured in siALDH2 EC lysates (48 h posttransfection). Data are presented as the mean ± SD of % NADH production vs. siCTR. ^∗^*p* < 0.05 and ^∗∗^*p* < 0.01 vs. siCTR. (d) Morphology of confluent monolayers of siCTR and siALDH2 B ECs fixed and stained 48 h posttransfection. A representative image for each condition is shown. Scale bar: 100 *μ*m. (e) ALDH2 activity measured in ECs in the presence or absence of daidzin (1–10 *μ*M). D10: 10 *μ*M daidzin. Data are presented as the mean ± SD of % NADH production vs. Ctr. ^##^*p* < 0.01 vs. Ctr.

Whereas in siCTR cells ALDH2 protein expression was easily detected by Western blot, the enzyme expression was drastically reduced in siALDH2 (Figure 1(a)). Silencing of ALDH2 also reduced the mRNA levels and activity of the enzyme (Figures 1(b) and 1(c)) and promoted a significant change in HUVEC morphology, characterized by the irregular elongated shape instead of the regular polygonal shape of siCTR cells (Figure 1(d)). As expected, 10 *μ*M daidzin significantly reduced ALDH2 activity, while at 1 *μ*M, it did not affect the enzyme activity (Figure 1(e)).

**Figure 2:**
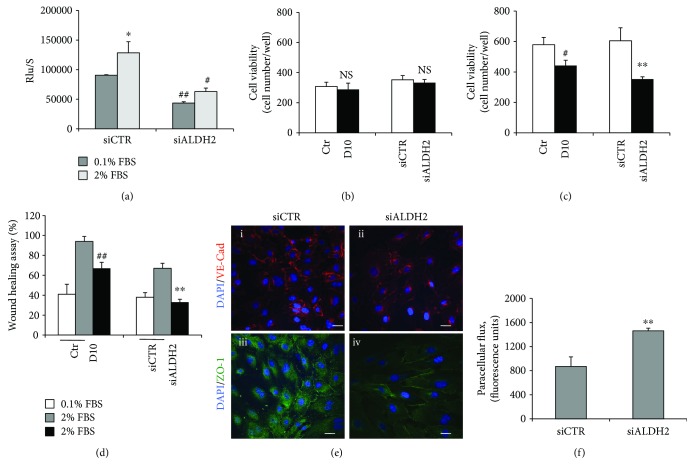
ALDH2 silencing or inhibition impairs endothelial functions. (a) BrdU incorporation in ECs transfected with siRNA for 24 h. Proliferative capacity was assessed after 48 h treatment with 0.1%–2% FBS. Data are reported as mean ± SD. ^∗^*p* < 0.05 vs. siCTR with 0.1% FBS; ^#^*p* < 0.05 and ^##^*p* < 0.01 vs. siCTR. (b) Cell survival in 10 *μ*M daidzin-treated ECs or siCTR and siALDH2 ECs exposed to 2% FBS for 2 and (c) 5 days. Data are expressed as means ± SD of the cell number counted/well. Dimethyl sulfoxide (DMSO) is used as a solvent to dissolve daidzin. No significant effect of DMSO was observed in HUVEC survival (DMSO-treated cell numbers/well: 329 ± 30). NS: not statistically significant; ^#^*p* < 0.05 vs. Ctr; ^∗∗^*p* < 0.01 vs. siCTR. D10: 10 *μ*M daidzin. (d) Scratch assay in 10 *μ*M daidzin-treated ECs or in siCTR and siALDH2 ECs cultured in 0.1% or 2% FBS for 18 h. Means ± SD of % of scratch closure (^##^*p* < 0.01 vs. Ctr; ^∗∗^*p* < 0.01 vs. siCTR). (e) Confocal analysis of VE-cadherin and ZO-1 patterns in control (i–iii) and siALDH2 ECs (ii–iv) after exposure to EBM-2 with 0.1% FBS for 8 h. Representative images of three experiments at 63x magnification are shown. Scale bar: 20 *μ*m. (f) Permeability in siCTR and siALDH2 ECs detected as fluorescence-conjugated FITC-dextran diffusion through the confluent monolayers after exposure to EBM-2 with 0.1% FBS for 8 h. ^∗∗^*p* < 0.01 vs. siCTR. Images are representative of results obtained with siALDH2 B.

Next, we determined the involvement of ALDH2 activity on cell viability, proliferation, and migration, as well as on cell permeability, key features of endothelial cell functions. The proliferation of siALDH2 ECs, assessed by the BrdU incorporation assay, was significantly reduced relative to siCTR ECs and not recovered in the presence of serum (Figure 2(a)). Accordingly, both deficiency and pharmacological inhibition of ALDH2 in HUVECs resulted in a consistent reduction of cell viability, particularly visible after 5 days (Figures 2(b) and 2(c)). Moreover, cell migration, evaluated by the scratch assay, was significantly inhibited in both daidzin-treated and siALDH2 ECs (Figure 2(d)). ALDH2 ablation also affected the adherence and tight junctions of HUVECs altering their barrier function. Indeed, in confluent siCTR, the expression of VE-cadherin and ZO-1, examined by immunofluorescence, was mainly localized at cell-cell contacts and disappeared in cells whose ALDH2 was silenced (Figure 2(e)).

To corroborate the immunofluorescence analysis, we evaluated the paracellular flux in a confluent monolayer of siCTR and siALDH2 ECs. Consistent with the above data, we observed an increase in permeability in siALDH2 when compared to siCTR ECs, as shown by increased paracellular flux of fluorescence-conjugated dextran (Figure 2(f)).

**Figure 3:**
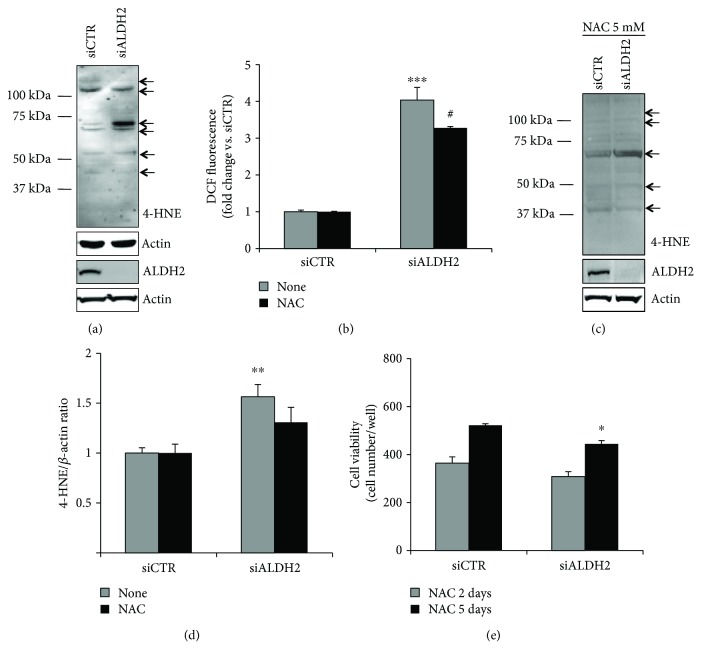
ALDH2 silencing increases 4-HNE protein adducts and ROS levels. (a) Western blot analysis of 4-HNE protein adducts in siCTR and siALDH2 ECs cultured in 2% FBS for 24 h. Knockdown efficiency was checked using immunoblotting with an ALDH2 antibody. The arrows indicate bands quantified in (d). Representative blots of at least 3 with similar results are shown. (b) ROS production in siCTR and siALDH2 ECs cultured in 0.1% FBS for 24 h in the presence/absence of NAC (5 mM). Cells were pretreated for 30 min with NAC before FBS treatment. Data, normalized for the cell number, are expressed as the mean of fold change ± SD vs. siCTR of DCF fluorescence. ^∗∗∗^*p* < 0.01 vs. siCTR; ^#^*p* < 0.05 vs. untreated siALDH2 ECs. (c) Western blot analysis of 4-HNE protein adducts in siCTR and siALDH2 ECs cultured in 2% FBS for 24 h with or without pretreatment (30 min) with NAC (5 mM). Knockdown efficiency was checked using immunoblotting with an ALDH2 antibody. The arrows indicate bands quantified in (d). Representative blots of 3 with similar results are shown. (d) Quantification of major bands (indicated with arrows), normalized to actin, is reported as a fold increase ± SD of ADU vs. siCTR. ^∗∗^*p* < 0.01 vs. siCTR. (e) Cell survival in siCTR and siALDH2 ECs exposed to 2% FBS in the presence/absence of NAC (5 mM) for 2 (grey bars) or 5 (black bars) days. Data are expressed as means ± SD of the cell number counted/well. ^∗^*p* < 0.05 vs. siCTR. Images are representative of results obtained with siALDH2 B.

Of note, silencing the ALDH2 triggered the accumulation of 4-HNE-induced covalent adducts, as well as ROS products [24] in ECs (Figures 3(a) and 3(b)), suggesting that the intracellular abundance of these toxic products might contribute to the impairment of the endothelium and trigger the premature senescence process seen below.

To get insight into the role of ROS in the dysfunction of ECs whose ALDH2 was silenced, 5 mM NAC, a ROS scavenger, was used. In particular, as shown in Figures 3(b)–3(e), NAC treatment in siALDH2 ECs affected the pattern of 4-HNE protein adducts and reduced their expression, significantly inhibited the production of total ROS levels, and improved viability at 5 days. Taken together, these results indicate that ALDH2 silencing affects the healthy phenotype of HUVECs by increasing the accumulation of 4-HNE-induced adducts and ROS products, leading to changes in morphology and disassembling of intercellular junctions, and impairs endothelial cell barrier function and mobilization capacity.

### 3.2. ALDH2 Silencing or Inhibition Promotes the Onset of Senescence in Endothelial Cells

**Figure 4:**
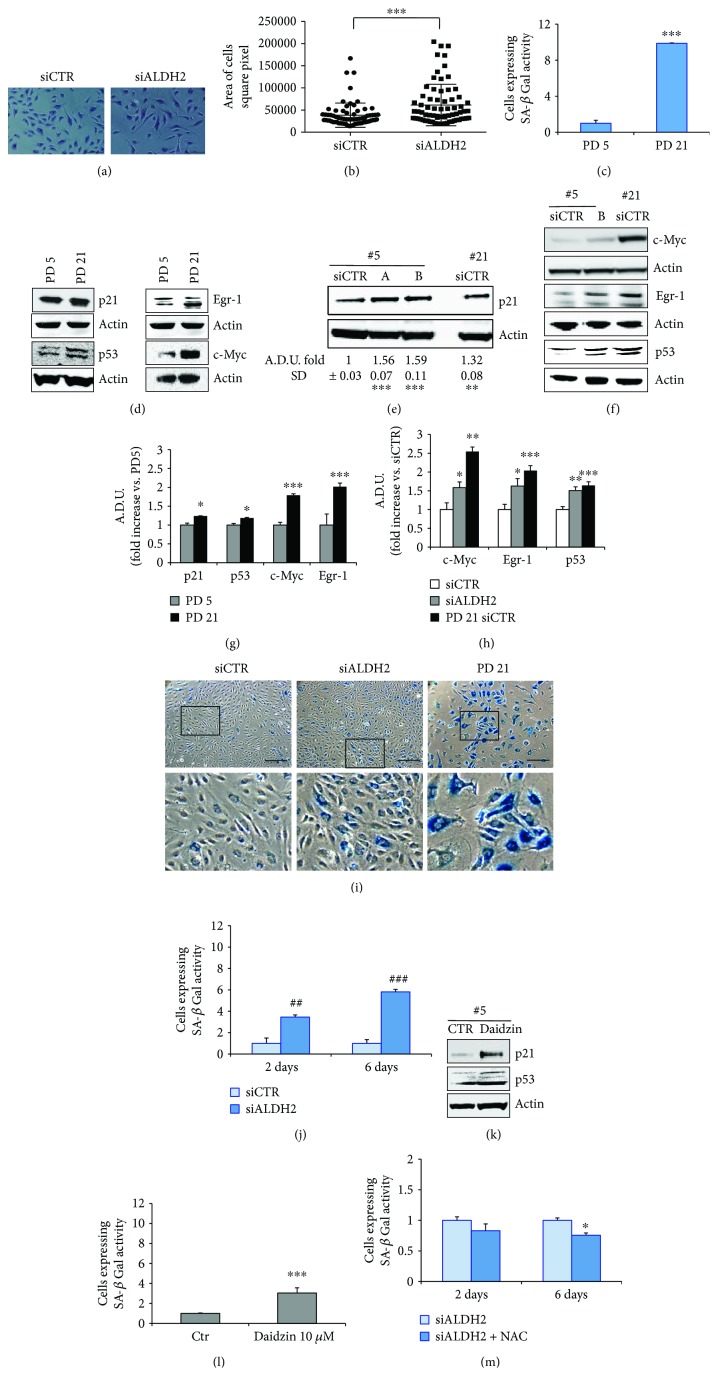
ALDH2 silencing or inhibition induces the expression of senescence markers in HUVECs. (a, b) Images and area of cells in siCTR and siALDH2 ECs cultured in EBM-2 supplemented with 2% FBS for 48 h. Data are expressed as a square pixel of cells analyzed using ImageJ. Quantification of 70 cell areas for each condition is reported. ^∗∗∗^*p* < 0.001 vs. siCTR. Two-way ANOVA was used. (c) SA-*β*-Gal quantification, expressed as a fold increase in positive cells for SA-*β*-Gal activity ± SD vs. PD 5. ^∗∗∗^*p* < 0.001 vs. PD 5. (d, e, f) Western blot analysis of a pattern of senescent markers (d, left: p21 and p53 or right: Egr-1 and c-Myc) in HUVECs at PD 5 (#5, PD 5) and PD 21 (#21, PD 21) or (e, p21 or f c-Myc, Egr-1, and p53) in siCTR and siALDH2 HUVECs, 48 h posttransfection (#5, PD 5; #21, PD 21). Representative blots of 3 with similar results are shown (e, g, h). Quantification of immunoblot in (d), (e), and (f). Data are reported as an ADU fold increase vs. siCTR (e, h) or vs. PD 5 (g). (e) ^∗∗∗^*p* < 0.001 and ^∗∗^*p* < 0.01 vs. siCTR. (g) ^∗∗∗^*p* < 0.001 and ^∗^*p* < 0.05 vs. PD5. (h) ^∗∗∗^*p* < 0.001, ^∗∗^*p* < 0.01, and ^∗^*p* < 0.05 vs. siCTR. (i) Images of SA-*β*-Gal staining of siCTR and siALDH2 ECs and PD 21 groups obtained with a Leica DMI4000 microscope. Images of HUVECs at PD 21 were reported as a positive control. Scale bar: 250 *μ*m. The insets show boxed areas in detail. (j) Cells were transfected with siRNA for 2 or 6 days. The transfection was repeated every 72 h. SA-*β*-Gal quantification, expressed as a fold increase ± SD vs. siCTR of positive cells for SA-*β*-Gal activity. ^##^*p* < 0.01 and ^###^*p* < 0.001. (k) Western blot analysis of senescent markers in HUVECs at PD 5 in the presence/absence of daidzin (10 *μ*M) for 48 h (#5, PD 5). Representative blots of 3 with similar results are shown. (l) SA-*β*-Gal quantification in ECs treated or not with daidzin (10 *μ*M) for 48 h, expressed as a fold increase in positive cells for SA-*β*-Gal activity ± SD vs. untreated cells. ^∗∗∗^*p* < 0.001 vs. untreated cells. (m) Cells were transfected with siRNA for 2 or 6 days in the presence/absence of NAC (5 mM). The transfection was repeated every 72 h. Cells were pretreated for 30 min with NAC, before the treatment with 2% of FBS. The pretreatment with NAC and the treatment with 2% FBS were repeated every 3 days. SA-*β*-Gal quantification, expressed as a fold decrease ± SD vs. untreated cells positive for SA-*β*-Gal activity. ^∗^*p* < 0.05. Images are representative of results obtained with siALDH2 B.

In light of the above findings, showing a pervasive dysfunction of endothelial cells upon ALDH2 silencing, we wondered whether this impaired state might be associated with the acquisition of premature senescence. Morphological modifications characterize usually senescent cells in *in vitro* culture: cells become large, flat, vacuolated, and occasionally multinucleated [25]. Therefore, we quantified the size of the cells and reported it as the area of the cells expressed as square pixel. siALDH2 cells presented a larger cell area in comparison to siCTR cells (Figures 4(a) and 4(b)). We next measured the cumulative population doubling (PD) in long-term cultured endothelial cells at predetermined set points, an experimental protocol we devised to study senescence [16]. We performed measurements of PD and of several senescence markers at PD 5 and PD 21, corresponding to the time-dependent progress from premature to moderate-full senescent cells, respectively. Senescent cells vary from other nonproliferating cells by several markers. Besides the expression of signals belonging to the two major senescence-inducing pathways (p53/p21, c-Myc, Egr-1), we also evaluated beta-galactosidase (SA-*β*-Gal) activity. As shown in Figures 4(c), 4(d), and 4(g), HUVECs at PD 21 overexpressed SA-*β*-Gal activity and senescence markers compared to HUVECs at PD 5. Of note, in HUVECs at PD 5 whose ALDH2 was silenced, we observed the overexpression of p53 and p21, c-Myc, and Egr-1 (Figures 4(e), 4(f), and 4(h)).

In addition, evaluation of SA-*β*-Gal revealed significant differences in its expression between siCTR PD 5 cells and those of siALDH2 groups and PD 21 groups (see Figure 4(i)). A time-related increase in SA-*β*-Gal expression (3- to 5-fold difference) was evident in siALDH2 but not in siCTR cells (Figure 4(j)). Similarly, treatment with 10 *μ*M daidzin significantly increased senescence markers in PD 5 ECs (Figures 4(k) and 4(l)). Of note, 5 mM NAC partially reversed SA-*β*-Gal expression in siALDH2 ECs (Figure 4(m)), corroborating the instrumental role played by ROS in the impairment of the endothelium whose ALDH2 was silenced.

These data clearly indicate that ALDH2 silencing and inhibition are associated with the onset of early signs of a senescent phenotype.

### 3.3. ALDH2 Silencing Alters Bioenergetic Functions in Endothelial Cells

**Figure 5:**
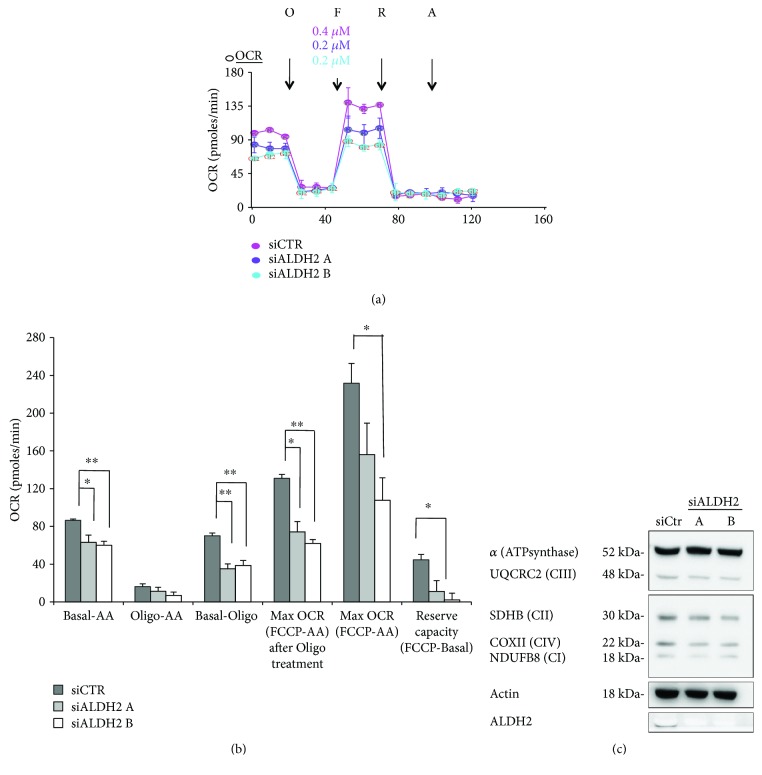
ALDH2 silencing is associated with mitochondrial dysfunction. (a) OCR was assessed by a Seahorse XF24 cell culture microplate in siCTR and siALDH2 ECs that were harvested and seeded 24 h posttransfection in XF24 cell culture plates at a density of 3 × 10^4^ cells/well. Where indicated (arrows), oligomycin (O) (1 *μ*g × ml^−1^), FCCP (F) (0.2–0.4 *μ*M), rotenone (R) (1 *μ*M), and antimycin A (AA) (1 *μ*M) were added. Data are representative of three experiments. (b) Basal OCR, proton leak, ATP-linked OCR, maximal OCR, and reserve capacity in siALDH2 ECs exposed to 2% FBS for 24 h. The means ± SEM of each parameter are shown. ^∗∗^*p* < 0.01 and ^∗^*p* < 0.05 vs. siCTR. (c) Western blot analysis of OXPHOS representative complexes detected by the OXPHOS antibody cocktail kit in siALDH2 (two clones A and B) or siCTR cultured in 2% FBS for 24 h. Knockdown efficiency was verified with an ALDH2 antibody. Actin was used as a loading control. Blots are representative of 3 with similar results.

Adjusting metabolism to a quiescent state is central to normal vessel function [9, 10]. As we observed that ablation of ALDH2 causes an impairment of permeability leading to a senescent phenotype in HUVECs, we investigated whether ALDH2 would affect cell oxidative metabolism. We evaluated oxygen consumption rates (OCR) in siCTR compared to siALDH2 at baseline and in response to oligomycin (Oligo), fluoro-carbonyl cyanide phenylhydrazone (FCCP), or antimycin A (AA) and rotenone (R). We found that ALDH2 silencing reduced basal and maximal respiration and decreased the respiratory reserve capacity (Figures 5(a) and 5(b)). Importantly, Western blot analysis of constitutive respiratory complexes including CII, CIII, and CIV, known to be sensible to the ROS increase in mitochondria, indicated that ALDH2 did not alter the abundance of these respiratory complexes (Figure 5(c)).

**Figure 6:**
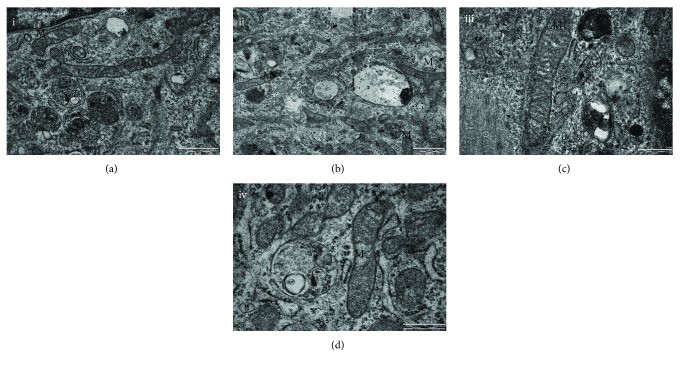
ALDH2 silencing affects the HUVEC mitochondrial ultrastructure. Cells were transfected with siRNA as described above. Then, they were harvested and seeded 24 h posttransfection and treated as described in Materials and Methods. TEM representative images of siCTR or siALDH2: (a, c) siCTR and (b, d) siALDH2. M: mitochondria. Scale bar: (a, b) 1 *μ*m and (c, d) 500 nm. Images are representative of results obtained with siALDH2 B.

Given the significant changes of the metabolic function reported, we assessed whether they were associated with morphological modification of cellular components. TEM images showed minor morphological changes in mitochondria of siALDH2 cells (Figure 6). In particular, smaller mitochondria in Figure 6(b) were observed when compared to those in Figure 6(a), and cristae in the centre of mitochondria in Figure 6(d) appear to be deleted.

In conclusion, bioenergetic analysis revealed that ALDH2 deficiency specifically reduced both mitochondrial respiration and reserve capacity, which presumably contribute to the reduction of the endothelial responsiveness.

## 4. Discussion

ALDH2, a mitochondrial enzyme, is known for its detoxifying properties, which provide living organisms with a protective shield against endogenous and exogenous toxic agents [26], such as acetaldehyde (alcohol metabolism) and products originating from lipid peroxidation (4-HNE) and ROS. The relevance of ALDH2 in providing strong protection toward toxic insults has been described in numerous reports, demonstrating its efficacy in various models of human diseases such as ischemia-reperfusion and ischemic stroke characterized by overwhelming oxidative stress [27]. Note that the above pathologies have the vascular endothelium as an underlying component whose function might be compromised by ROS and aldehyde surge.

Previous data from our group documented that alteration in endothelial function induced by A*β* was restored by the activation of mitochondrial ALDH2 in the endothelium [15]. However, the contribution of ALDH2 to endothelial senescence remains unresolved.

Here, we have described the role of ALDH2 in endothelial growth and function, using HUVECs as a model, whose ALDH2 was silenced through transfection of targeting siRNA or exposure to the pharmacological inhibitor of ALDH2, daidzin. The resulted cellular models displayed a number of morphological and functional changes. Morphologically, we observed a subverted phenotype of silenced cells, characterized by an enlarged and elongated cell shape, in sharp contrast to the polygonal one of wild-type endothelial cells. Functionally, the ALDH2 loss yielded a reduced mobility and augmented permeability, a finding consistent with the marked decline of VE-cadherin and ZO-1 expression at cell-cell contacts and with the changes in cell morphology. We also observed a reduction of cell proliferation that results in a reduction of the cell number. Predictably, ALDH2 silencing produced intracellular accumulation of 4-HNE adducts and ROS production, which appears to be the primary cause of the observed impairment of endothelial cell functions and morphological changes, as corroborated by using the scavenger NAC.

The study of mitochondrial respiration provided further insight into the mechanism underlying endothelial dysfunction, as mitochondria possess a considerable respiratory reserve, which is important in the response to oxidative stress [11, 12]. Indeed, we found that ALDH2 silencing diminished the inherent oxidative metabolism, as indicated by a decline in the oxygen consumption rate. Specifically, siALDH2 cells showed a reduction in basal and maximal respiration measured under basal conditions and in response to FCCP, with a clear decrease in the reserve capacity. While mitochondrial respiration decreased, the analysis of the expression of all respiratory complexes does not change upon ALDH2 downregulation, indicating that the protein level of all respiratory complexes is not the leading cause of reduced respiration in siALDH2 cells. We therefore suggested that the decrease in basal OCR and spare reserve capacity might be due to some posttranslational modifications.

Furthermore, TEM images suggest alterations in mitochondrial morphology of siALDH2 cells. Thus, the endothelial dysfunction, noted in siALDH2 cells and in ECs exposed to daidzin, may be attributed to the effects of accumulated toxic products and to subtle defects of mitochondrial respiration.

Investigation on endothelial cell senescence was initiated in view of the observed morphological similarities between siALDH2 and those typical of senescent cells, i.e., the flat morphology and enlarged cell size. Evidence sustaining the hypothesis of an incipient senescent state was gleaned from the analysis of SA-*β*-Gal activity and specific intracellular signals measured in siALDH2 cells and in daidzin-treated cells subjected to stress-induced senescence experiments in which population doublings (PD) were recorded. Indeed, cellular senescence ensued as early as at PD 5 progressing steadily up to PD 21. In fact, increases in signals, e.g., SA-*β*-Gal, p21/p53, Egr1, and c-Myc, were noted when comparing signalling patterns at PD 5 vs. PD 21. The onset of senescence in siALDH2 cells as well as in ECs exposed to daidzin is considerably faster than what was observed in the earlier work on HUVECs exposed to exogenous amyloid peptides (PD 5 vs. PD 21) [16]. This underscores the protective role of ALDH2 exerted toward the endothelium to an extent not appreciated before.

## 5. Conclusions

Our results demonstrate that in the vascular endothelium, loss of ALDH2 accelerates the acquisition of a premature senescence phenotype leading to endothelial dysfunction. These events are associated with an increase in ROS levels, accumulation of 4-HNE protein adducts, and impairment of mitochondrial bioenergetic functions. The senescence phenotype of the endothelium, with exhaustion of the regenerative capacity, may represent a defensive response from the damage caused by an accumulation of toxic aldehydes and can lead to the expansion of the senescent cell population further aggravating the loss of function in the vasculature.

## Data Availability

The data used to support the findings of this study are included within the article.

## Abbreviations

AA: Antimycin A
ALDH2: Aldehyde dehydrogenase 2
DAPI: 4′,6-diamidino-2-phenylindole
EGM-2: Endothelial growth medium
EBM-2: Endothelial basal medium
ECs: Endothelial cells
FCCP: Carbonylcyanide-p-trifluoromethoxyphenyl hydrazone
NAC: N-Acetyl-L-cysteine
PD: Cumulative population doubling
siALDH2: siRNA targeting ALDH2
siCTR: Scrambled control siRNA.
